# A study of potential laser-induced degradation in remote standoff Raman spectroscopy for wall paintings

**DOI:** 10.1140/epjp/s13360-022-03305-2

**Published:** 2022-10-01

**Authors:** Yu Li, Amelia Suzuki, Chi Shing Cheung, Yuda Gu, Sotiria Kogou, Haida Liang

**Affiliations:** Imaging and Sensing for Archaeology, Art History and Conservation (ISAAC) Lab, School of Science and Technology, Nottingham Trent University, Nottingham, NG11 8NS UK

## Abstract

A mobile remote standoff Raman spectroscopy system operational at typical distances of 10 m was developed specifically for research of historical sites and wall paintings recently. Here we present an upgrade to that system informed by a thorough experimental investigation of the relevant laser-induced degradation issues. Reflectance spectroscopy as a more sensitive technique than Raman spectroscopy was used for monitoring and a new phenomenon of reversible alterations was detected in many paint samples at very low laser intensities of less than 1 W/cm^2^ when Raman measurements detected no changes. Contrary to conventional wisdom, the intensity threshold for safe operation was found to decrease significantly for larger incident irradiation area in the case of a vermilion oil paint sample. Damage threshold in intensity for each material needs to be determined for different spot sizes, which can be orders of magnitude lower for 1 mm spot size compared with micro-Raman. Results from this study is also relevant to portable Raman systems which use similarly large spot sizes. However, the larger spot size still generates more Raman photons overall under safe operation than micro-Raman systems. Continuous-wave (CW) lasers are found to be best suited to efficient, that is more Raman signal detected over a given measurement time, and safe Raman operation than ns-pulse lasers at the same wavelength. While the damage threshold in intensity for ns-pulse lasers is much higher than that of CW lasers, the pulse energy allowed in one pulse for safe operation is still too low to allow detection of Raman signal, and the need for multiple pulses makes pulse laser inefficient owing to the low repetition rate necessary to ensure adequate heat dissipation between pulses. The safety of the upgraded system was evaluated and found that no permanent laser-induced degradation was detected within 60 s of laser irradiation for any of the paint samples.

## Introduction

Raman spectroscopy for material identification is highly specific and non-invasive if used with care, which makes it a popular technique for in situ analysis of cultural heritage assets. Over the last few decades, Raman spectroscopy has been successfully applied to a full range of research problems and materials in heritage, from art history to conservation and from the identification of materials on corrosion layers on glass and metals to pigment and paint. The field has been extensively reviewed over the years including some very recent reviews [1–4]. Within the cultural heritage science domain, instrument development has been focused on mobile Raman systems for in situ non-invasive analysis of heritage materials. However, drawbacks in their practical use, in particular for wall paintings and large monuments, such as difficulty in positioning the probe, instability (vibrations) of the scaffold in supporting sensitive measurements that needs integration times greater than a few milliseconds, and the inefficiency in re-positioning the system to access different parts of a large monument have been noted [5]. Recently, we reported the development and application of a remote standoff Raman system at working distance of 3–15 m dedicated to research of historical sites and large paintings and monuments [6, 7]. The system can be placed at one position on the ground and take measurements of any part of an architectural interior from difficult to access recesses in sculptures to ceiling paintings at lofty heights. It is necessary to clarify what we mean by remote standoff Raman spectroscopy as there is confusion on the meaning of ‘remote’ between various disciplines within cultural heritage science research and between the wider Raman community and the remote sensing community. For example, ‘remote’ can sometimes mean ‘non-contact’, so that mobile Raman operation, where the probe is a few cm away from the object, is considered ‘remote’; or that the probe is on a scaffold placed next to a wall painting but connected with a long fibre to the rest of the Raman system or computer is considered ‘remote’. In the remote sensing community, ‘remote’ usually means that the entire instrument including the probe is at a large distance from the object, e.g., in satellite and airborne remote sensing. Within heritage science, the term ‘standoff’ is usually used for imaging systems placed a couple of metres away from the object. To avoid confusion, here we use the term “remote standoff” to describe our Raman system, where the entire instrument is placed on the ground at > 3 m from the object with typical working distance of the order of 10 m.

Prior to its practical application in situ, one of the concerns in heritage research using laser-based techniques is the risk of laser-induced degradation to heritage assets. The statement of Raman being non-invasive needs qualifying, as the laser intensity (power per unit area) could be high enough to cause alteration to materials even with a relatively low laser power of 0.01–1 mW for a typical micro-Raman system with laser spot size of a few microns [8, 9]. This is particularly important in the context of large area Raman analysis such as Raman mapping. And in the case of our recently developed macro-Raman mapping technique using the remote standoff Raman system where even larger areas were probed [7].

For laser-induced degradation effects, special care needs to be taken in heritage research to make sure conservation ethics are taken into consideration. However, only a few systematic studies have been carried out in this field, such as on pigments and paints [8, 10–13], and currently, the safe use of Raman spectroscopy usually depends on the operator’s experience only. Finally, the concern is not only limited to laser safety for the materials, but also on the accurate identification of the original materials if the damage causes changes in the molecular structure and thus changes in Raman signals.

In Raman microscopy studies, visual observation through the microscope is often used to verify whether any damage is induced [9, 14, 15]. However, subtle degradation can be difficult to perceive visually even under a microscope [16]. Sometimes Raman spectra themselves are used to confirm degradation [9, 11, 13, 17] by monitoring the occurrence of new peaks, peak shifts or broadening, or abrupt changes in peak intensity owing to molecular alterations or the formation of new phases. However, any alteration due to the initial Raman measurement used for comparison with subsequent Raman measurements would not have been detected; and the amount of materials altered, if any, may be below the detection limit of the Raman system. Raman scattering efficiency is orders of magnitude (~ 10^6^) lower than that of diffuse scattering measured by reflectance spectroscopy. Thorough investigation using techniques more sensitive to material changes is necessary to evaluate laser-induced degradation in Raman spectroscopy.

In this paper, a systematic study of laser-induced degradation effects on selected common paint samples is conducted to inform an upgrade of our remote standoff Raman system and guide the safe use of this system in cultural heritage research.

## The upgraded remote standoff Raman system

**Fig. 1:**
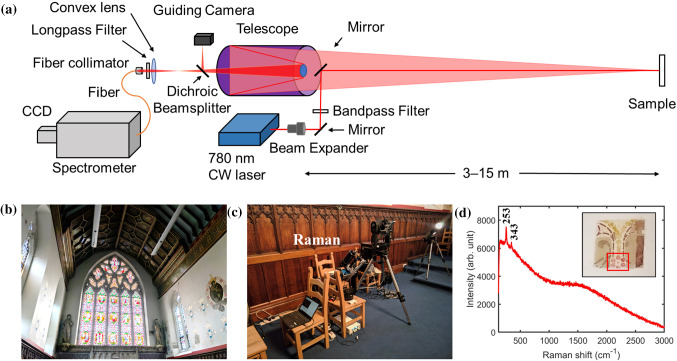
**a** Schematic of the upgraded standoff Raman system with a focused laser beam; **b** the full view of the chapel of the Convent of Mercy where the wall on the right was analysed; **c** the mobile remote standoff Raman system deployed in situ; **d** Raman spectrum collected from a distance of ~ 6 m on the original paint layer revealed in the cleaning test area (see picture in the inset)

The original configuration of this system operational at 3–15 m has been described in detail in our previous publications [6, 7]. The system employed a 780 nm continuous-wave (CW) laser as excitation source. The laser beam was co-axial with the telescope optical axis. The output beam was considered as collimated at the aperture with a divergence of 0.5 mrad, which resulted in a spot size on the target of ~ 4 mm at 4 m distance. The maximum laser power at target corresponds to an irradiance of 0.36 W/cm^2^. The advantage of using a collimated beam is to avoid refocusing the beam at different distances. However, the spot size would exceed 1 cm at distances greater than 10 m. To achieve higher spatial resolution and better efficiency, the mobile standoff Raman system has been redesigned and upgraded to include a 5 × beam expander which is used to focus the beam at different distances (Fig. 1a). The spot diameter of the focused beam at target is ~ 1 mm. The maximum laser power at sample gives an irradiance of ~ 5.5 W/cm^2^. The spectral resolution is ~ 4 cm^−1^ over the spectral range of 140–1300 cm^−1^ recorded with a 1200 lines/mm grating, or ∼8.5 cm^−1^ over 140–3300 cm^−1^ with a 500 lines/mm grating.

Figure 1b-d shows an example of in situ deployment of the remote standoff Raman system for analysis of wall paintings in the chapel of the Convent of Mercy associated with the Cathedral Church of St Barnabas in Nottingham, which was designed and built in 1846 by the pioneering Gothic Revival architect Augustus Welby Pugin [18]. Measurements were carried out in a cleaning test area (at a height of ~ 4 m) where the surface overpaint layers have been removed, revealing the decoration underneath. The distance between the position where the standoff Raman system was deployed and the wall paintings were over 6 m. Figure 1d shows an example Raman spectrum collected from the red area of the original paint layer identified with vermilion.

## Laser-induced degradation tests

Discussions in our earlier publication [6] show that the efficiency advantage of CW versus pulsed lasers for Raman detection depends on the damage threshold. Efficiency of Raman detection here is defined to be Raman signal detected for a given measurement time, or by proxy, the incident laser energy on the material for a given measurement time. Given the more stringent requirements for measurements to be non-invasive in heritage material analysis, it was thought that remote standoff Raman spectroscopy for heritage science should use CW lasers unlike other remote Raman spectroscopy applications (e.g., explosives detection) where pulsed laser was typically used [19–21]. It is worth noting that pulse lasers are employed in hybrid laser-based systems involving Raman spectroscopies for cultural heritage applications recently [22–24]. However, there is limited data on the damage threshold comparisons for CW and pulsed lasers on relevant materials. To better understand laser-induced degradation effects on common pigments and paints, laser irradiation experiments were designed using three types of laser sources to test the differences between pulsed and CW lasers at the same wavelength of 532 nm and between CW lasers at different wavelengths at 532 nm and 780 nm. Since larger laser spot sizes are employed in remote standoff Raman systems than those of micro-Raman systems typically used in cultural heritage research, the effects of spot size were also investigated in addition to that of intensity and fluence. We use the term ‘fluence’ for both pulse and CW lasers.

### Experimental setup and procedure

**Fig. 2:**
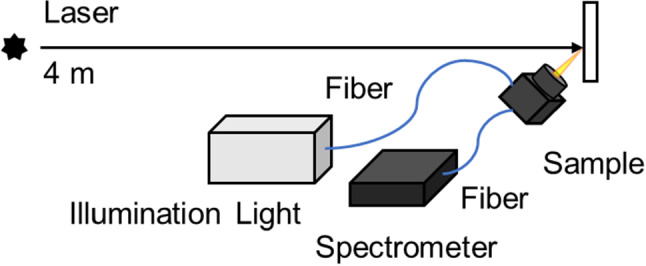
Experimental setup for measuring laser-induced degradation effect in the paint samples

A combined setup of a laser source and the fibre optic reflectance spectroscopy system as shown in Fig. 2 was used to investigate the laser degradation effects. All experiments were conducted at a distance of 4 m between the laser used and the test panel. The laser beam irradiated the vertically placed sample at normal incidence, while a retroreflective probe was aligned at 45° angle to the laser beam to collect reflectance spectra before and after the laser irradiation with an elliptical spot of 0.45 mm by 0.75 mm within the irradiated area of the sample. A low power tungsten halogen light (Mikropack DH-2000) was used as the illumination source for reflectance spectroscopy. The reflectance calibration was performed against a Labsphere Spectralon white standard. Reflectance spectra were collected by a spectrometer (Ocean Optics HR2000) with an integration time of 200 ms, averaged for 10 times, amounting to 2 s for each measurement. To ensure stable light output, the tungsten halogen light was warmed up for 1800 s before taking measurements. The stability of the illumination source was tested prior to the experiments by continuously monitoring the reflectance changes of the white standard until it stabilises, which usually takes 1800 s and the tungsten light was left on throughout the experiments.

Three reflectance spectra were recorded successively (every 2 s) for each sample before laser irradiation and a consecutive series of reflectance spectra were collected over a period of 6–300 s straight after the irradiation. To make sure that no other factors interfered with the degradation tests, a set of control measurements were taken each time before the degradation tests, using an identical procedure but without the laser switched on. Difference in reflectance spectra (ΔR) in the visible range (400–900 nm) for all samples were monitored by subtracting the reflectance spectrum before the laser irradiation from the spectra collected after the irradiation.

Three types of lasers were used as irradiation source for laser-induced degradation tests: a 780 nm CW, a 532 nm CW and a 532 nm pulsed laser to compare the effects of CW lasers at different wavelengths and pulsed versus CW lasers on painting materials. The 780 nm CW laser is the one employed in our mobile remote standoff Raman spectroscopy system, which is a temperature stabilised external cavity diode laser with a maximum output power of 90 mW and power stability ~ 1% over 1 h. The 532 nm CW laser is a temperature controlled diode-pumped solid-state (DPSS) laser with a power stability of ~ 1% over 1 h. The 532 nm pulsed laser was generated by the second harmonic of a 1064 nm Q-switched Nd:YAG source (Continuum Minilite ML II) with pulse duration of ~ 5 ns, maximum repetition rate of 15 Hz and pulse to pulse energy stability better than 3% (rms stability of 1%). The tests always started from a low pulse energy. If no degradation was observed, the energy would be increased. Laser irradiation was always limited to around ~ 1 mm^2^ on the sample, irrespective of the actual size of the laser beam and thus allowing for adjustment of the incident laser intensity through varying the beam size without changing the size of the illuminated area. The lasers all have Gaussian-like beam profile.

A total of 12 common historic artists pigments of a range of colours and reflectance spectra were selected to be examined for laser-induced degradation risks. The samples are linseed oil based paints made with the pigments and painted on plywood boards with a preparation layer of chalk in rabbit skin glue and have been naturally aged for 15 years [25]. Basic information of the pigments can be found in Table 1. A summary of the results is shown in Table 2.

**Table 1:** Pigments used to prepare the reference paints for laser-induced degradation tests

Pigment	Main composition	Supplier
Red lead	Pb_3_O_4_	Kremer Pigmente
Vermilion	HgS	Beijing Jinbizhai pigment plant
Vermilion light	HgS	Kremer Pigmente
Cochineal lake	Mexican cochineal dyestuff on an alumina substrate	National Gallery London
Realgar (grade 3)	AsS	Beijing Jinbizhai pigment plant
Chrome yellow (medium)	PbCrO_4_	Kremer Pigmente
Lead white	PbCO_3_Pb(OH)_2_	Kremer Pigmente
Ultramarine (synthetic)	Na_6–10_Al_6_Si_6_O_24_S_2–4_	Kremer Pigmente
Indigo	C_16_H_10_N_2_O_2_ Indigo and silicaceous extender	Kremer Pigmente
Prussian blue	Fe_4_[Fe(CN)_6_]_3_·nH_2_O (n = 14–16) Hydrated iron hexacyanoferrate complex	Beijing Jinbizhai pigment plant
Azurite (grade 3)	2CuCO_3_·Cu(OH)_2_ Copper (II)-carbonate	Kremer Pigmente
Verdigris (synthetic)	Copper-(II)-acetate	Kremer Pigmente
Malachite	CuCO_3_·Cu(OH)_2_	Kremer Pigmente

**Table 2:** Laser-induced alterations on oil paints

Pigment	Laser	Alteration^1^	Dwell time (s)	Intensity^2^ (W/cm^2^)	Fluence (J/cm^2^)
Azurite	532 nm pulsed	*N*	5 × 10^–9^	6 × 10^7^	0.3
	532 nm pulsed	***Y***	5 × 10^–9^	1 × 10^8^	0.6
	532 nm CW	*N*	1800	0.3	5 × 10^2^
	532 nm CW	***Y***	10	3.5	35
	780 nm CW	*N*	1800	5.5	1 × 10^4^
Red lead	532 nm pulsed	*N*	5 × 10^–9^	3 × 10^6^	1.5 × 10^–2^
	532 nm pulsed	R*	10 × 5 × 10^–9^	3 × 10^6^	1.5 × 10^–1^
	532 nm pulsed	***Y***	5 × 10^–9^	3 × 10^7^	1.5 × 10^–1^
	532 nm CW	*N*	10	0.1	1
	532 nm CW	***R***	10	0.3	3
	780 nm CW	*N*	10	0.3	3
	780 nm CW	***R***	10	5.5	55
	780 nm CW	***R***	360	5.5	2 × 10^3^
Realgar	532 nm pulsed	*N*	5 × 10^–9^	4 × 10^4^	2 × 10^–4^
	532 nm pulsed	***Y***	5 × 10^–9^	4 × 10^5^	2 × 10^–3^
	532 nm CW	*N*	10	9 × 10^–2^	0.9
	532 nm CW	***Y***	10	0.3	3
	780 nm CW	***R***	10	0.3	3
	780 nm CW	***R***	10	5.5	55
	780 nm CW	***R/Y***	360	5.5	2 × 10^3^
Vermilion	532 nm pulsed	*N*	5 × 10^–9^	4 × 10^4^	2 × 10^–4^
	532 nm pulsed	***Y***	5 × 10^–9^	1 × 10^5^	6 × 10^–4^
	532 nm CW	*N*	10	0.1	1
	532 nm CW	***R/Y***	10	0.3	3
	780 nm CW	*N*	10	0.4	3
Vermilion light	780 nm CW	***R***	10	0.3	3
	780 nm CW	***R***	10	5.5	55
	780 nm CW	***R***	360	5.5	2 × 10^3^
Verdigris	532 nm pulsed	*N*	5 × 10^–9^	1 × 10^8^	0.6
	532 nm CW	*N*	10	0.8	8
	532 nm CW	***R***	10	3	30
	780 nm CW	***R***	1800	0.3	6 × 10^2^
	780 nm CW	*N*	60	5.5	3 × 10^2^
	780 nm CW	***Y***	1800	5.5	1 × 10^4^
Chrome yellow	780 nm CW	*N*	10	0.3	55
	780 nm CW	***R***	10	5.5	55
	780 nm CW	***R***	60	5.5	3 × 10^2^
	780 nm CW	***R***	360	5.5	2 × 10^3^
Prussian blue	780 nm CW	*N*	300	5.5	2 × 10^3^
Cochineal	780 nm CW	*N*	60	5.5	3 × 10^2^
	780 nm CW	***Y***	360	5.5	2 × 10^3^
Lead white	780 nm CW	*N*	60	5.5	3 × 10^2^
Ultramarine	780 nm CW	*N*	60	5.5	3 × 10^2^
Indigo	780 nm CW	***R***	60	5.5	3 × 10^2^
Malachite	780 nm CW	*N*	60	5.5	3 × 10^2^

In all cases, ~ 1 mm^2^ of the sample was illuminated regardless of the laser spot size

^1^ N*—*no alteration; *R—*reversible alteration; *Y—*alteration without reversion

^2^ In case of pulsed laser, the intensity of a single pulse is given

^*^Just noticeable alteration followed by reversion; 10 pulses delivered at 1 Hz repetition rate

### Sensitivity of Raman spectroscopy for monitoring laser-induced degradation effects

First, we examine the adequacy of Raman spectroscopy as a tool for monitoring laser-induced alteration, since Raman spectra has sometimes been used to monitor the potential degradation induced by the Raman laser. This method is still open to debate as few studies on the relation between the laser intensity, fluence, and the changes of Raman spectral features have been performed. Burgio et al. [8] observed new peak formation along with peak shift and decrease of intensity of the peaks, which clearly identified degradation products induced by CW lasers during Raman analysis of lead containing pigments above an intensity threshold. Philippidis et al. [13] reported the observation of abrupt decrease of Raman spectra when darkening occurred on vermilion pigment powder without a binder. But in other studies, such as De Santis et al. [9], Raman spectroscopy failed in the detection of laser-induced degradation of indigo when damage was clearly visible from microscopic images. This is not surprising as Raman scattering efficiency is only 1 in 10^5^ to 10^7^. Reflectance spectroscopy is many orders of magnitude more sensitive than Raman spectroscopy, and we used it previously to examine the degradation of pigments caused by a Nd:YAG ns-pulsed laser at 1064 nm and shown to be highly effective [26]. Our combined setup of Raman and reflectance spectroscopy allows simultaneous interrogation of degradation effects at the same area to evaluate to what extent Raman spectroscopy can be used to monitor laser-induced degradation.

**Fig. 3:**
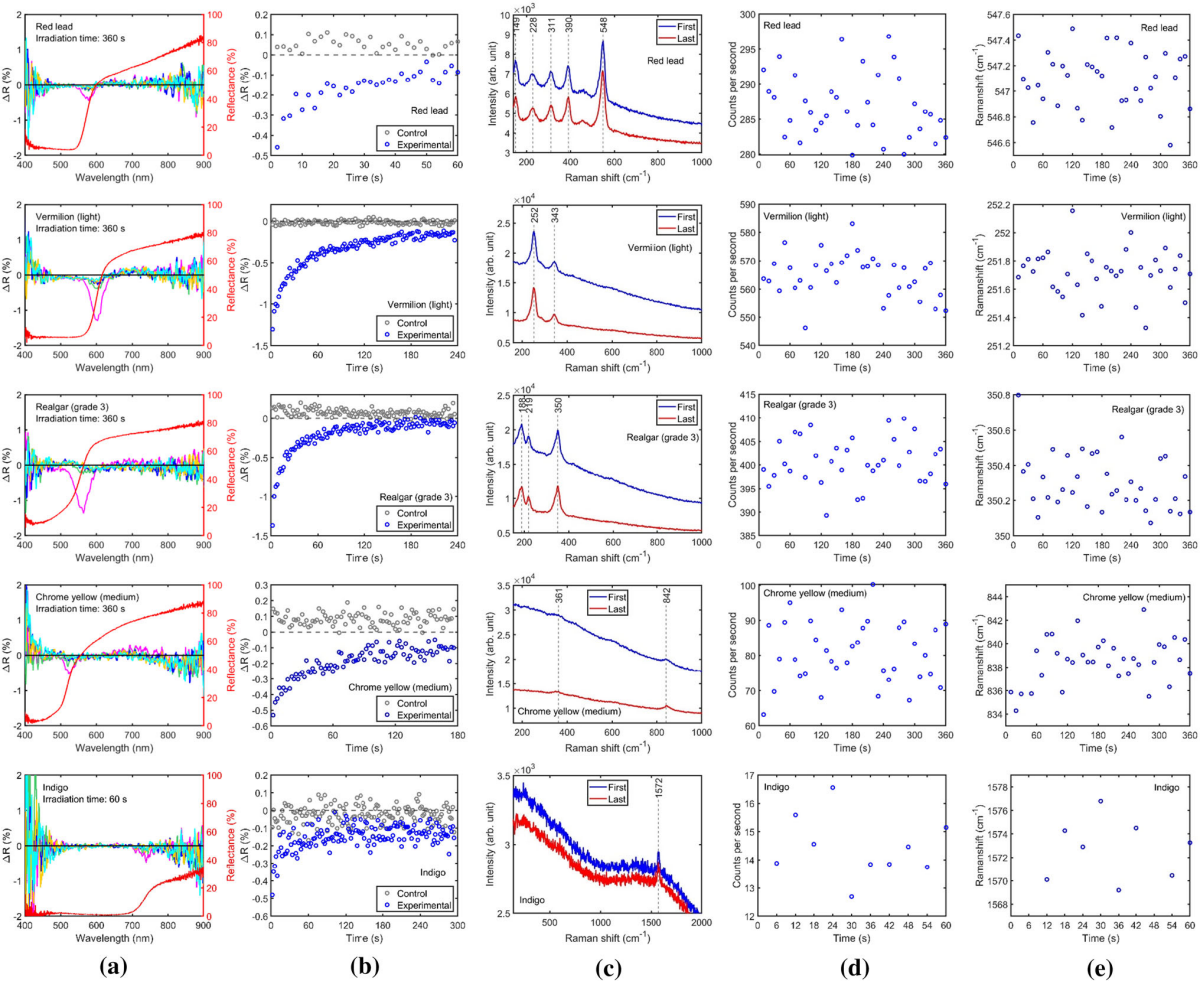
Reflectance and Raman evolution results of laser irradiation on oil paint samples using focused 780 nm beam (1 mm diameter): **a** change in reflectance spectra (ΔR) and the original reflectance of the paints (red curve), five ΔR spectra are plotted at intervals of 72 s (for red lead, vermilion light, realgar, and chrome yellow) and 12 s (for indigo) in order of time as follows: magenta, green, blue, yellow, and cyan; **b** time evolution of the magnitude of the dip in ΔR after laser irradiation (blue data points) compared with control measurement without laser irradiation (grey data points); **c** Raman spectra comparing the first and last spectra collected during laser irradiation; **d** time evolution of the net peak intensity of the strongest Raman peak for each pigment (548 cm^−1^ for red lead, 252 cm^−1^ for vermilion light, 350 cm^−1^ for realgar, 842 cm^−1^ for chrome yellow, and 1572 cm^−1^ for indigo); **e** time evolution during Raman measurements of the positions of the strongest Raman peak for each pigment, determined by Gaussian fitting of the peaks

Raman measurements were conducted during the 60–360 s laser irradiation tests for oil paints made from 5 different pigments: red lead, vermilion (light), realgar, chrome yellow and indigo (Table 2 and Fig. 3). The Raman data were collected either as a set of 36 consecutive spectra with 10 s integration time each (36 × 10 s) or a set of 10 spectra with 6 s integration time each (10 × 6 s). The evolutions of net peak counts per second of the Raman peaks above the continuum spectrum, are plotted in Fig. 3d which show no changes in Raman intensity. The Raman peak intensities were obtained by subtracting a linear baseline fit around the peak using 5 data points either side of the peak on the continuum part of the spectrum free from Raman signal. Similarly, no shifts were found in the evolution of the Raman peak position by fitting 5 datapoints around the peak (Fig. 3e). The spectrometer was calibrated using mercury-argon or neon gas discharge calibration lamps first to measure the wavelength of the lasers before adjusting the grating and recalibrating the spectrometer for Raman measurement by making sure that the Rayleigh scattering line is outside the measured spectral range. Reflectance spectra were collected before and after Raman measurements and found to be altered by Raman laser irradiation in all samples. However, the alteration was transient, and they all reverted towards the initial spectrum during the 60–360 s monitoring time post-irradiation. Temporal alteration in reflectance spectra followed by reversion was first reported in our recent conference paper [7]. Evolution plots of reversions in reflectance spectra were demonstrated by monitoring the maximum dip in the ΔR spectra (Fig. 3b). Detection of alteration by reflectance spectroscopy but not by Raman, suggests that either Raman spectroscopy could not provide sufficient sensitivity to detect minor changes or the temporal changes in reflectance did not correspond to alteration in the crystalline structure of the pigment (e.g., alteration in the binding medium). It is also worth noting that no visual changes were observable. Colour changes, ΔE_00_, can be calculated [27] from the reflectance spectra before and after laser irradiation, assuming CIE standard D65 illumination and the 1931 standard 2 degrees observer. It was found to be well below 1 and therefore not noticeable even under a microscope in almost all the cases. The only exception was in the case of realgar where the 532 nm CW laser caused a permanent change corresponding to a colour change ΔE_00_ of 1.3.

### CW versus pulse laser at 532 nm

As discussed in our previous paper [6], there is a balance between safe and efficient Raman measurements, which determines whether it is optimal to use a pulsed or CW laser for safe and efficient remote standoff Raman measurements. Here the relative efficiency of Raman measurement is defined by the total laser energy incident on a material, which is proportional to the Raman signal generated for a given material, in the same measurement time. The ratio of the measurement times between using a CW laser and a pulsed laser at the same wavelength with the same spot size is given by

1 $$\frac{{t_{cw} }}{{t_{p} }} = \frac{{f_{p} R_{p} }}{{I_{cw} }}$$

where $I_{cw}$ is the CW laser intensity damage threshold at the sample, $f_{p}$ is the damage threshold in fluence for one pulse and $R_{p}$ is the pulse repetition rate for the pulsed laser. For a given material below its damage threshold, the total laser energy delivered is proportional to the Raman signal.

We chose the laser wavelength of 532 nm for this comparison because both CW and pulsed lasers are readily available at this wavelength. In this study, the ns-pulsed laser with pulse duration of ~ 5 ns delivers at least 5 orders of magnitude higher intensity in one pulse than the CW laser, while the CW laser delivers 2–4 orders of magnitude more energy in 10–1800 s irradiation time than a single ns-pulse. CW lasers can have a measurement advantage of 6–8 orders of magnitude in efficiency for safe operation below the damage threshold, when compared with a typical ns-pulsed laser operating at a repetition rate of 1–100 Hz and pulse duration of ∼5 ns, if the damage threshold in laser intensity (power per unit area) are at the same level for CW and pulsed lasers. However, this assumption is not true as can be seen in Table 2 where the intensity threshold for degradation is 6–8 orders of magnitude higher for pulsed laser than CW laser. This is most likely because the heat generated in a pulse can be dissipated between the pulses.

**Fig. 4:**
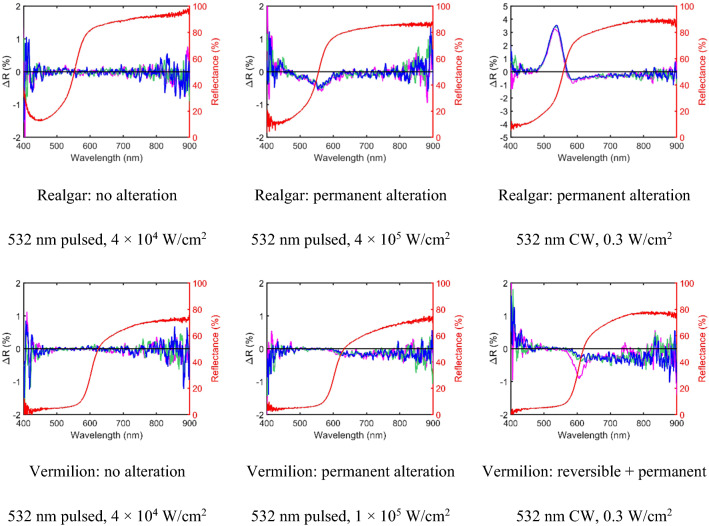
Degradation results of realgar and vermilion using 532 nm pulsed and CW irradiation: the original reflectance (red curve) and 3 reflectance difference plots immediately after laser irradiation in 2 s interval (in order of time: magenta, green, and blue)

Using the above Eq. (1) for the ratio in measurement time between CW and pulsed laser, we observe that for the most sensitive pigments, realgar and vermilion, where the damage threshold is of order of 0.2 mJ/cm^2^ in one pulse and of order 0.1 W/cm^2^ with the CW laser at irradiation time of 10 s, CW mode is more efficient in Raman measurements even compared with a pulsed laser with 100 Hz repetition rate. For red lead where the damage threshold is of the order of 15 mJ/cm^2^ in one pulse and 0.1 W/cm^2^ with the CW laser at 10 s irradiation time, the efficiency of the CW laser is comparable to the pulsed laser at 10 Hz repetition rate. For the pigments that are more resistant to laser induced alteration, such as azurite and verdigris, where the damage threshold is greater than 300 mJ/cm^2^ in one pulse and of order 1 W/cm^2^ with the CW laser at 10 s irradiation time, the pulse laser is more efficient at 10 Hz repetition rate. For all 5 pigments examined, the CW mode is more efficient than the pulsed mode if repetition rate is kept at 1 Hz. Figure 4 shows some of the laser-induced degradation results comparing 532 nm pulsed lasers at different intensities with the 532 nm CW laser. It is noteworthy that the 532 nm CW laser-induced alteration in vermilion appears to consist of a transient alteration at ~ 600 nm and a permanent alteration at longer wavelengths.

The above equation overestimates the Raman efficiency using the pulsed laser, since it assumes that if one pulse does not cause damage, then accumulation of multiple pulses would not cause damage either. This was examined experimentally on red lead where a just noticeable but reversible alteration was observed after 10 pulses at a repetition rate of ~ 1 Hz, when no alteration was observed after one pulse at the same intensity of $3\times {10}^{6}$ W/cm^2^ (Table 2). It is also interesting to note that one pulse at 10 times greater intensity caused permanent alteration. The above shows that as expected, the damage threshold for delivering more than one pulse will be lower than that of just one pulse at the same intensity. Increasing repetition rate of pulse lasers will not necessarily improve their efficiency in safe Raman detection, because the higher the repetition rate the less time for heat dissipation and the lower the damage threshold. This damage threshold will eventually approach the CW value as the repetition rate is increased. The experimental results confirm our previous decision to select a CW laser for our remote standoff Raman system as it is more efficient for safe Raman analysis of wall paintings.

### CW lasers at 532 nm versus 780 nm

The difference in laser induced damage between 532 and 780 nm CW lasers was investigated, and it was found that the damage threshold for 780 nm is greater than 532 nm for all 5 paints examined (azurite, red lead, realgar, vermilion and verdigris). Either no alteration or less alteration (e.g., reversible rather than permanent alteration) was observed when using 780 nm laser at the same or higher intensity and fluence than the 532 nm laser. This is perhaps not surprising for red lead, realgar and vermilion, since all have the typical ‘S’ shaped spectrum of semiconductors with point of inflection at > 532 nm which means high absorption of light at 532 nm and low absorption at 780 nm. However, both azurite and verdigris have similar reflectance at the two wavelengths and neither have an absorption band at these wavelengths, but the shorter wavelength of 532 nm still has a lower damage threshold (Table 2). It was found in one of our previous studies [26], materials that are transparent have the highest damage threshold and materials that are either highly absorbing or highly scattering have lower damage threshold. Absorption is not the only mechanism that determines the damage threshold.

### Effect of spot size

The remote standoff Raman uses a laser spot size of order 1 mm which is much larger than those used in standard benchtop Raman systems (a few to tens of microns). Can the Raman intensity threshold for laser induced degradation learnt from these conventional systems be applicable to remote standoff Raman? We compared two sets of measurements on vermilion (light) oil paint with incident laser spot sizes of 0.88 mm and 0.04 mm and found that the intensity threshold for safe operation (i.e. no alteration including reversible ones) is at least 90 times higher for the smaller spot size than the larger one, which means that the system with the larger spot size is at most 5 times more efficient under safe operation below damage threshold compared with the one using the smaller spot size, if we take the total laser energy incident at the sample to be proportional to the Raman signal and assuming the instrument efficiency to be the same for the two setups. With a larger spot size, more total energy will be delivered on the material for the same beam intensity, which makes heat dissipation more difficult than that of a smaller area. Conventional wisdom of just considering intensity damage threshold is not sufficient, as that would have assumed that the thresholds are the same for both spot sizes and therefore the one with the larger spot would be able to safely gather ~ 480 times more Raman photons. Raman damage threshold in intensity and fluence need to be evaluated for different spot sizes.

Other recent studies [13, 28] also found that the laser damage threshold increases with decreasing spot size. Philippidis et al. [13] found that the damage threshold is higher by a factor of ~ 10 between spot sizes of 26 and 162 μm respectively in their analysis of vermilion pigment powder, which means one can safely collect up to ~ 4 times more Raman photons with the larger spot compared with the smaller one. Note that the damage threshold in that study is defined by a sharp drop in Raman intensity which occurs at a much higher laser intensity and is a permanent damage. Nevertheless, the conclusions on the effect of spot size are consistent with our measurements on vermilion (light) oil paint.

### 780 nm CW laser-induced degradation effects on a range of common pigments

**Fig. 5:**
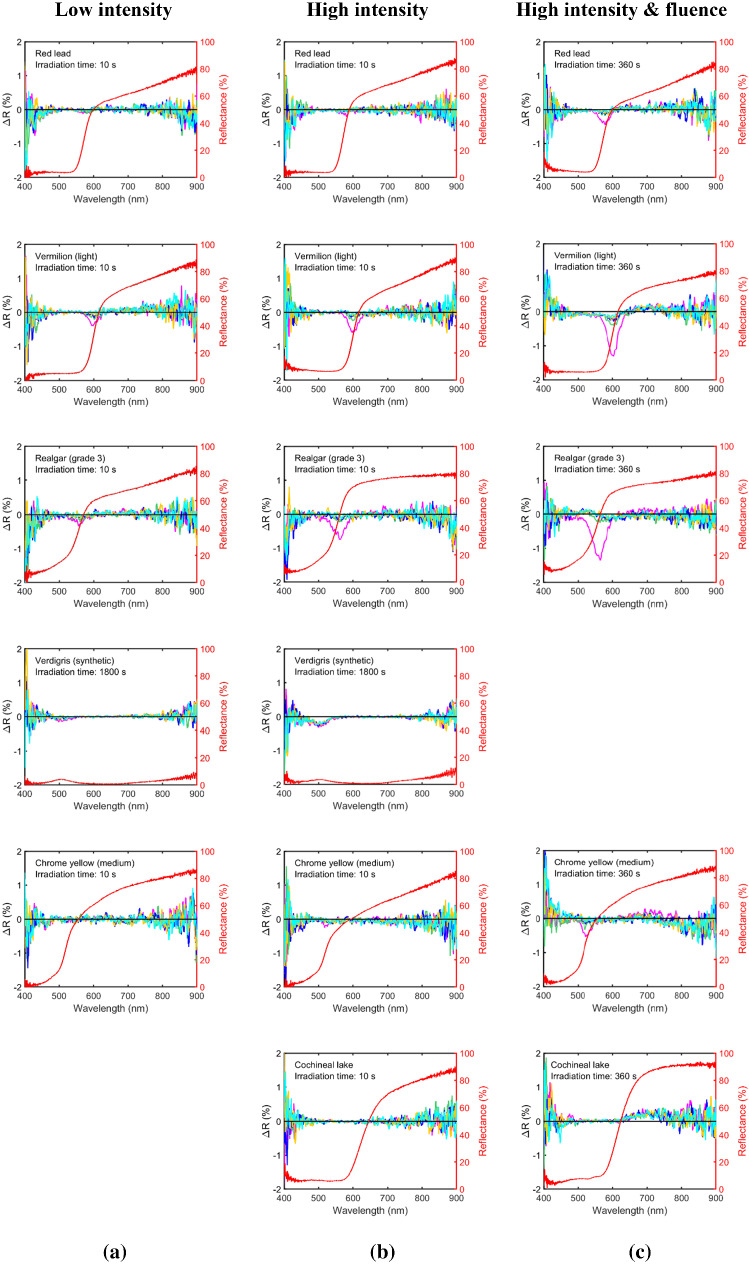
Laser irradiation of pigments with 780 nm CW laser: **a** when using collimated beam at intensity of 0.3 W/cm^2^; **b** focused beam at intensity of 5.5 W/cm^2^ with the same irradiation time as in (**a**); and **c** focused beam at intensity of 5.5 W/cm^2^ at a longer irradiation time of 360 s and therefore higher fluence compared to (**b**). The original reflectance spectrum (red curve) and 5 reflectance difference spectra immediately after laser irradiation in equally divided time intervals (in time order: magenta, green, blue, yellow, and cyan)

The upgraded 780 nm remote standoff Raman system uses a focused beam of ~ 1 mm diameter spot at the sample for all distances and operates at a maximum intensity of 5.5 W/cm^2^. A range of 13 historic artist’s oil paint samples were selected, including organic and inorganic pigments with different colours and spectral shapes (Table 1, 2) to verify the safe use of the upgraded remote standoff Raman system on wall paintings. A comparison between incident laser intensities of 0.3 W/cm^2^ corresponding to the original system and 5.5 W/cm^2^ corresponding to the upgraded system was made while keeping the size of the irradiated area similar (Fig. 5). Longer irradiation times were also tested for those with low Raman scattering efficiency.

No alteration was detected at the higher intensity of 5.5 W/cm^2^ for Prussian blue, azurite and cochineal. In all other pigments, a more significant alteration was observed at the higher intensity of 5.5 W/cm^2^. Reversible alteration was observed for red lead, realgar, vermilion (light) and chrome yellow, even with 10 s irradiation time or fluence of 55 J/cm^2^; the alteration stayed reversible at longer irradiation times of 360 s (Fig. 5). Longer time was also required for ΔR to fully revert after 360 s irradiation compared with 10 s irradiation, which may be the result of heat accumulation resulting from higher laser fluence. Furthermore, the magnitude of the dip in ΔR was significantly larger than those with only 10 s irradiation (Fig. 5). Overall, the largest change in reflectance tend to occur at the wavelength where the reflectance spectra have the sharpest features.

It is interesting to note that those samples that have high Raman scattering efficiency (red lead, realgar, vermilion and chrome yellow) experience only reversible alteration under laser intensity of 5.5 W/cm^2^ and fluence between 55 and $2.0\times {10}^{3}$ J/cm^2^. While those with low Raman scattering efficiency such as azurite and verdigris behave differently. No alteration was detected in azurite even after 1800 s irradiation at the higher laser intensity of 5.5 W/cm^2^, reaching a fluence of ${10}^{4}$ J/cm^2^. Alterations of a more permanent nature were observed in both verdigris and cochineal only at longer irradiation times of 1800 s and 360 s, respectively (Table 2 and Fig. 5). Whether the degradation effect was permanent for verdigris and cochineal remains to be examined, as the monitoring time after the irradiation might not be long enough to cover a possible slow reversion process. Overall, if a laser intensity of 5.5 W/cm^2^ is used, a total irradiation time of 60 s will not cause a permanent alteration for any of the pigments studied in Table 2.

## Conclusions

The upgraded 780 nm remote standoff Raman spectroscopy setup with a focused beam of 1 mm at target and maximum intensity of 5.5 W/cm^2^ is shown to be safe under 60 s of continuous laser irradiation without permanent alteration for all 13 common historic artists’ paints investigated. Of these, no alteration was detected in azurite, verdigris, Prussian blue, cochineal, lead white, and ultramarine, while reversible alteration was detected in red lead, realgar, vermilion, chrome yellow and indigo. This latter group have relatively high Raman scattering efficiency and typical integration time needed in Raman spectroscopy is much less than 60 s. Raman spectroscopy did not detect laser-induced alteration that are easily detectable by reflectance spectroscopy. Either these subtle alterations are not the result of changes in crystalline structure of the material, or Raman spectroscopy, as a technique that is orders of magnitude less sensitive than reflectance spectroscopy, was not able to detect the changes. Future work will explore the nature of the laser-induced transient and permanent alterations observed in this study. Laser-induced alteration threshold is found to be strongly dependent on the size of the irradiated area such that the threshold can be orders of magnitude higher for smaller spot size, therefore the upgrade from collimated to the focused beam with elevated laser intensity is not expected to affect the safe operation of the Raman system significantly. However, there is much to gain from the higher spatial resolution and constant spot size independent of working distance offered by the focused beam in the upgraded system. The choice of a CW laser for remote standoff Raman spectroscopy in cultural heritage applications was justified based on experimental determination of alteration thresholds of each material using a CW laser and a nanosecond pulsed laser at the same wavelength of 532 nm. Raman measurements using CW laser is always more efficient, that is shorter measurement time required for the same Raman signal collected, under safe operational limits. The alteration threshold in intensity was found to be higher or similar at 780 nm compared with 532 nm CW laser for all materials tested, which combined with the reduction of fluorescence at 780 nm favoured the used of the 780 nm laser in the remote standoff Raman system. This study explored a laser intensity and spot size regime that is orders of magnitude different from micro-Raman spectroscopy commonly used in the study of cultural heritage materials, demonstrating that thresholds for safe Raman operations in intensity and fluence needs to be re-examined for different spot sizes. Recent adoption of portable Raman systems in the field shares similar intensity and spot size as the remote standoff Raman system described in this study and caution needs to be exercised in their operation since the intensity thresholds can be orders of magnitude lower compared with micro-Raman systems.

The upgraded remote standoff Raman spectroscopy system has been adopted as part of the mobile lab research infrastructure for heritage science on offer to the European cultural heritage science research community and beyond. This study informed the upgrade decisions and guides the safe operation of the system for heritage materials.

## Data Availability

This manuscript has associated data in a data repository. [Authors’ comment: The datasets generated during and/or analysed during the current study are available from the corresponding author on reasonable request.]
